# Too Young to Gamble: Long-term Risks from Underage Gambling

**DOI:** 10.1007/s10899-024-10319-1

**Published:** 2024-05-29

**Authors:** Levi Pérez

**Affiliations:** Department of Economics, Jovellanos Faculty of Commerce, Tourism and Social Sciences, University of Oviedo, Luis Moya Blanco, 261, 33203 Gijón, Spain

**Keywords:** Gambling Starting Age, Underage, Youth Gambling Behavior, Problem Gambling, Conditional Analysis, Ordered Probit

## Abstract

The increasing availability of gambling opportunities worldwide has the potential to impact youth gambling behavior and the prevalence of gambling-related harm. This paper studies whether starting gambling at an early age (i.e., below the minimum legal gambling age) increases an individual’s likelihood of suffering some kind of gambling-related harm in the future. Data taken from the *Study on the Prevalence, Behavior and Characteristics of Gamblers in Spain* provide information on respondents’ gambling starting age and assess gambling risk based on criteria established by the Diagnostic and Statistical Manual of Mental Disorders (DSM-IV). Estimates from an ordered probit model suggest that starting to gamble while under the minimum legal age is associated with a higher likelihood of problem gambling.

## Introduction

Gambling—i.e., wagering on uncertainty—is becoming increasingly popular as an alternative form of leisure and entertainment. This trend has been accompanied by a growing highly competitive supply. Many young people are currently growing up in societies where gambling is present through a range of technological, social, and print media (Kristiansen et al., 2017). This increased availability of gambling opportunities has the potential to impact youth gambling behaviors, including the prevalence of problem gambling among young people. Although legal age limits usually exist in regulated gambling environments, research suggests that underage gamblers often get around the law (Welte et al., 2009). Age has long been considered in the literature as a potential factor when analyzing gambling prevalence and risk factors for problem gambling (Mok & Hraba, 1991).

Previous studies indicate that age has an inverse U-shaped relationship with gambling expenditure, with expenditure rising from youth to middle age, peaking in middle age, and declining thereafter (Díaz & Pérez, 2021; Okunna et al., 2016; Perez & Humphreys, 2011). Overall, younger generations have a higher conditional probability of starting gambling (Díaz et al., 2023) and existing literature reports the general prevalence and perception of gambling among university students (Hira & Monson, 2000; Shin & Montalto, 2015; Wickwire et al., 2007). Delfabbro et al. (2009) conclude that although many adolescents experiment with gambling, few become regular gamblers in adulthood. The likelihood that an individual will engage in gambling falls with age (Humphreys & Pérez, 2012).

Findings have been mixed, however, as to whether there is an association between youth gambling and problem gambling in adulthood. Research identifies a number of individual risk factors and correlations between gambling and problem gambling among young people (Shead et al., 2010). Granero et al. (2014) suggest that age plays a significant role in moderating the likelihood of pathological gambling problems. The prevalence of pathological gambling is particularly high among youth as compared to adults (Volberg et al., 2010). Indeed, studies show that rates of problem gambling are up to five times higher among youth than adults (Kristiansen et al., 2015; Shaffer et al., 1999). Volberg (1994) and Lynch et al. (2004), among others, argue that most adults with problematic gambling behavior start gambling prior to adulthood; problematic youth gambling is often associated with starting to gamble at an early age (Burge et al., 2004).

Despite being banned for minors in most jurisdictions, gambling products are extensively advertised on television and social media platforms. Consequently, there is legitimate concern about whether this increasing popularization and availability of gambling has the potential to harm children and adolescents (Brevers et al., 2022). Rahman et al. (2012) find that starting gambling from an early age is associated with gambling-related harms in adolescent Internet gamblers. Similarly, an early gambling onset age may be predictive of adult problems including substance use disorders and other psychiatric concerns (Jiménez-Murcia et al., 2010; Lynch et al., 2004). Vitaro et al. (2004) show that earlier- and later-onset gamblers had different gambling behavior patterns suggesting that prevention strategies should be group-specific. Increased gambling-related harm has been described for adolescents in many jurisdictions including Australia (Miller, 2017), Canada (Elton-Marshall et al., 2016), Croatia (Ricijas et al., 2016), New Zealand (Volberg et al., 2010), Sweden (Fröberg et al., 2015), the United Kingdom (Gambling Commission, 2017), and the United States (Marchica & Derevensky, 2016).

This paper aims to analyze whether starting gambling at an early age (particularly under the legally allowed age) increases an individual’s likelihood of being classified in a risk gambling risk group, as is suggested in the previous literature. To examine this question, data were collected from a cross-sectional survey that provides information on the age at which respondents began gambling and includes a number of questions to assess risky gambling behaviors in Spain. Beyond being a pioneering study in the Spanish context, this study is novel in that it focuses on whether the social problem of underage gambling truly exists and if there are any associated long-term risks (gambling-related harms). Even though it is not possible properly to establish causal effects in the current study due to the nature of the data and the empirical approach followed, the results may be useful in identifying correlations and have practical implications in terms of policies regulating legal gambling age.

## Methods

### Data and Variables

The Spanish gambling prevalence survey (DGOJ, 2015) provides a nationally representative dataset of the Spanish population. It consists of 6,816 individuals aged 18 (the legal age for gambling) and over who answered a questionnaire regarding sociodemographic and gambling characteristics. The survey provides information on respondents’ age, gambling starting age, and gambling behavior and it is part of the Spanish Government’s responsible gambling strategy, which began in 2013 with the goal of minimizing the potential risks of gambling through prevention and the protection of the most vulnerable groups.

Of the respondents, individuals who reported having never gambled were removed from the sample, as were observations with encoding errors. Individuals who failed to respond to key variables—most of whom did not remember the age at which they started gambling—were also removed from the sample. Accordingly, 1,983 individuals (29% of the sample) were dismissed, and the final sample for analysis consisted of 4,833 respondents.

Prior research consistently reports that problem gambling is strongly affected by socio-demographic factors (Layton & Worthington, 1999) and the consumption of alcohol and other substances (Ida & Goto, 2009). In general, gambling disorders are usually associated with being young, male, lower-educated, and/or a smoker or alcohol user (Wardle et al., 2011). Gambling problems among youth, in particular, are often associated with poor school performance, as well as alcohol, tobacco, and drug use (Winters et al., 1993). Accordingly, exogenous factors that may affect individuals’ attitudes towards gambling and, therefore, the prevalence of at*-*risk and problem gambling, such as current age, gender, marital status, employment status, education level, and alcohol and tobacco consumption, were considered in the analysis. Data on gambling products for which the individual reported gambling at least once were also observed in order to test whether some gambling products may be associated with risky or problematic gambling, as well as to control for the type of gambling consumption.

Descriptive statistics are presented in Table 1. The average age of respondents was 48 years old. The average starting age for gambling was approximately 23 years. Some individuals who self-identified as gamblers started gambling under 18, claiming to have placed their first bet as young as 4 years old. It should be noted that while the legal gambling age in Spain is 18, it does not seem to prevent minors from gambling.

**Table 1:** Summary statistics of the variables (obs = 4,833)

Variable	Mean	SD	Min	Max
Gambling starting age	22.847	8.250	4	75
*Sociodemographics*				
Age	48.440	16.733	18	94
Gender (male = 1)	0.506	0.500	0	1
Married	0.596	0.491	0	1
Employee	0.504	0.500	0	1
Higher education	0.230	0.421	0	1
Smoking	0.353	0.478	0	1
Drinking alcohol	0.762	0.426	0	1
*Gambling to…*				
Lottery	0.942	0.234	0	1
Scratch cards	0.211	0.408	0	1
Football pools	0.260	0.439	0	1
Slots	0.111	0.314	0	1
Casino	0.067	0.251	0	1
Bingo	0.172	0.378	0	1
Sports betting	0.041	0.198	0	1

Gender, marital status, and employment were evenly distributed among the sample, but just 23% of individuals reported having higher education. Lifetime cigarette smoking and alcohol use were coded as either “yes” or “no” depending on whether the respondents had ever tried either substance. The most popular gambling product in Spain was by far the lottery; over 94% of individuals in the survey reported having ever played the lottery.

As it did not seem very realistic that individuals began gambling at the age of 4 (as reported in the survey data), the variable representing the age at which the individual reported starting to gamble is coded in intervals. Two age groups are considered for the age at which individuals started to gamble (Table 2): below 18 years old (minimum legal gambling age) and at 18 years of age or older (reference group).

**Table 2:** Gambling starting age (obs = 4,833)

Range	Freq	Percent	Cum
< 18	699	14.46	14.46
> 18	4,134	85.54	100

The survey also included a number of questions to assess gambling risk based on criteria established by the Diagnostic and Statistical Manual of Mental Disorders (DSM-IV). This screening allows individuals to be grouped into four classifications of gambling severity: no-risk gamblers, low-risk gamblers, moderate-risk gamblers, and problem gamblers (Table 3). The percentage of no-risk gamblers (Group 1) was 89.30%, while 2.15% of self-reported gamblers had a likely diagnosis of pathological, or problem gambling (Group 4).

**Table 3:** Gambling severity groups (obs = 4,833)

Gambling severity group	Freq	Percent	Cum
No risk	4,316	89.30	89.30
Low risk	324	6.70	96.01
Moderate risk	89	1.84	97.85
Problem gambling	104	2.15	100

### Econometric Modelling

The dependent variable is an ordinal categorical variable that places each individual in a gambling severity group, ordered from lowest (Group 1) to highest risk (Group 4). Accordingly, an ordered probit model was estimated to explore the relationship between an individual’s starting gambling age and the risk that the individual would suffer some kind of gambling-related harm (i.e., the probability of being included in a particular gambling severity group), conditioned on a set of socioeconomic and demographic controls.

1 $$Pr\;\left(gambling\;severity\;group=i\right)=Pr\left(K_{i-1}<\beta_1\;gambling\;starting\;age<18+\beta_2\;age+\beta_3\;gender+\beta_4\;married+\beta_5\;employee+\beta_6\;higher\;education+\beta_7\;smoking+\beta_8\;drinking+\beta_9\;lottery+\beta_{10}\;scratch\;cards+\beta_{11}\;footballpools+\beta_{12}\;slots+\beta_{13}\;casino+\beta_{14}\;bingo+\beta_{15}\;sports\;betting+u_j\leq K_i\right)$$

where *gambling severity group* represents the categorical dependent variable (no-risk, low-risk, moderate-risk, and problem gambling). The independent variable *gambling starting age* accounts for the age at which the individual self-reported first gambling. *Age*, *gender*, *married* (whether the individual is married or not), *employee* (whether the individual is employed or not), and *higher education* (whether the individual has higher education) reflect the individual’s socio-demographic characteristics. *Smoking* and *drinking* are categorical/binary variables that identify whether the individual is a tobacco and/or alcohol user. *Lottery*, *scratch cards*, *football pools, slots*, *casino*, *bingo*, and *sports betting* are binary variables controlling for the gambling activities in which the individual participates; coded as 1 if a respondent has participated in an activity and zero otherwise. The error term u_j_ is assumed to be normally distributed.

The model specification allows for the estimation of the coefficients, β_1_, β_2_…, β_15,_ together with the cut points K_1_, K_2_…, K_I−1_, where I = 4 is the number of possible outcomes (no-risk, low-risk, moderate-risk, and problem gambling).

## Results

Estimates from the ordered probit model are shown in Column (I) of Table 4. There is a statistically significant link between the gambling starting age and the likelihood of being classified in an at-risk gambling group. Gambling before 18 years of age (legal gambling age) has a stronger positive correlation with the probability of being classified in a risk group, compared to starting to gamble at 18 years or older and above.

**Table 4:** Results dependent variable is gambling severity group (obs = 4,833)

Variable	(I)	(II)
Gambling starting age (reference group is > 18)		
< 18	0.211***	
Gambling starting age (reference group is > 40)		
< 15		0.353***
15–17		0.272***
18–24		0.106
25–39		0.067
*Socio-demographics*		
Age	0.005***	0.006***
Gender (male = 1)	0.155***	0.147**
Married	-0.215***	-0.217***
Employee	-0.026	-0.028
Higher education	-0.128*	-0.132**
Smoking	0.143***	0.145***
Drinking alcohol	0.048	0.044
*Gambling to…*		
Lottery	0.054	0.050
Scratch cards	0.201***	0.197***
Football pools	0.072	0.071
Slots	0.684***	0.683***
Casino	0.323***	0.326***
Bingo	0.215***	0.206***
Sports betting	0.464***	0.464***

Standardized beta coefficients; * *p*-value < 0.10, ** *p*-value < 0.05, *** *p*-value < 0.01

On average, for those individuals who report having started gambling before reaching 18, the probability of being classified in the no-risk group decreases by 3.3 percentage points, while the probability of being classified as a problem gambler increases by nearly 1 percentage point, from around 2% to 3% (Table 5). In other words, those who start gambling earlier than 18 years old have a 50% higher likelihood of problem gambling relative to those who start gambling after 18.

**Table 5:** Marginal effects of start gambling below 18 (obs = 4,833)

Gambling severity group	
No risk	-0.033***
Low risk	0.018***
Moderate risk	0.006***
Problem gambling	0.009***

^***^ *p*-value < 0.01

Regarding the control variables, the age of the individual at the time of the survey is associated with a greater risk of suffering gambling-related harm. A positive, statistically significant gender coefficient indicates that men exhibit higher odds of gambling-associated risk. In line with previous findings, being single is correlated with having higher problem gambling scores, so greater gambling risk, (Elton-Marshall et al., 2018) and smoking status is associated with riskier gambling behavior (Ida & Goto, 2009). Education status and gambling have a negative relationship. Those with higher levels of education are less likely to be classified in an at-risk group than those with a lower educational level, as predicted by previous studies (Coups et al., 1998).

The estimated coefficients for the gambling activities in which an individual participates show that slots, casino games, bingo, sports betting, and scratch cards are linked to a higher risk of gambling harm. Although it dominates gambling in Spain, the estimates presented in Table 4 suggest the lottery is not associated with problem gambling. While the empirical evidence on lottery playing and problem gambling is limited to the Spanish context, findings from Granero et al. (2023) are consistent with the impression that while most problem gamblers play the lottery, the lottery is their main gambling activity in only a small proportion of cases.

### Robustness Check

As the reference group includes those who reported starting gambling just after reaching the minimum gambling legal age, the sample was divided into five age groups to check the robustness of the model in measuring the effect of gambling as a minor (Table 6). Two new age groups (18–24; 25–39;) were added, and the under-18 group was split into two groups (< 15; 15–17) based on the age group structure of the survey. Those 40 years and older were classified as the new reference group.

**Table 6:** Gambling starting age (obs = 4,833)

Range	Freq	Percent	Cum
< 15	188	3.89	3.89
15–17	511	10.57	14.46
18–24	2,058	42.58	57.04
25–39	1,038	21.48	78.52
> 40	1,038	21.48	100

As shown in Column (II) of Table 4, a significant effect still exists for both the under-18 groups, while the 18–24 and 25–39 groups are not significantly different from the reference group. Starting to gamble under the age of 15 is positively correlated with the probability of future problem gambling, increasing by 1.4 percentage points compared to the reference group. For those who start gambling at 15–17 years old, the likelihood increases by 1.1 percentage points (Table 7).

**Table 7:** Marginal effects of gambling starting age (obs = 4,833)

Gambling severity group	< 15	15–17	18–24
No risk	-0.056***	-0.043***	-0.017
Low risk	0.031***	0.024***	0.009
Moderate risk	0.010***	0.008***	0.003
Problem gambling	0.014***	0.011***	0.004

^***^ *p*-value < 0.01

To test the consistency of the results, the model was re-estimated after dropping the observations of 16 individuals who reported starting gambling at a very young age (below 10 years old) from the sample. The estimated marginal effects do not change substantially. The increase in the probability of problem gambling for those who start gambling before the age of 15 increases to 1.6 percentage points, however (Table 8).

**Table 8:** Marginal effects of gambling starting age (obs = 4,817)

Gambling severity group	< 15	15–17
No risk	-0.060***	-0.043***
Low risk	0.033***	0.024***
Moderate risk	0.011***	0.008***
Problem gambling	0.016***	0.011***

Individuals who report having started gambling at a younger age than 10 years old were removed from the sample. *** *p*-value < 0.01

## Concluding Remarks

This paper contributes to the public debate regarding the consequences of expanded gambling opportunities on youth gambling behavior and the growing social concern about underage gambling. The data set used came from a cross-sectional retrospective survey in Spain that provides information on the gambling starting age of respondents and includes a number of questions to assess gambling risk based on criteria established by the DSM-IV. Based on a conditional analysis framework, an ordered probit model was used to empirically examine whether starting to gamble at an early age (i.e., before the legal gambling age) positively correlates with an individual’s likelihood of suffering from problem gambling (i.e., the probability of being included in a particular gambling severity group), when controlling for socioeconomic, demographic, and gambling factors.

Overall, starting to gamble before the age of 18 (i.e., the minimum legal gambling age in Spain) is positively associated with an increased probability of being classified in a gambling risk group. This is an interesting finding since public health concerns over gambling issues have been the strongest argument against the widespread expansion of gambling opportunities to date.

The results of this study may enhance regulators’ ability to understand gambling behaviors amongst subgroups of the population, including youth, and identify ways to improve gambling regulations and policies to reduce the potential harms associated with gambling. If evidence shows that individuals who gamble under the age of 18 are more likely subsequently to become problem gamblers, it may be possible to have more targeted interventions and support for youth.

## Data Availability

Data associated with the study is publicly available to download at https://www.ordenacionjuego.es/es/estudio-prevalencia.

## Appendix

Table 9

**Table 9:** Relevant questions from the survey

Variable	Question
Gambling starting age	At around what age did you first start playing a game involving financial stakes?
Age	Can you tell me your age?
Gender	Indicate gender
Married	Can you tell me your marital status?
Employee	Current employment status
Higher education	Educational level of the interviewee
Smoking	Do you currently smoke?
Drinking alcohol	Please tell me how often you drink wine, beer, spirits or any type of alcoholic beverages
Gambling to…	Could you please indicate whether you have participated in these gambling activities involving wagering money?
